# Nitro-Arachidonic Acid Prevents Angiotensin II-Induced Mitochondrial Dysfunction in a Cell Line of Kidney Proximal Tubular Cells

**DOI:** 10.1371/journal.pone.0150459

**Published:** 2016-03-04

**Authors:** Beatriz Sánchez-Calvo, Adriana Cassina, Natalia Rios, Gonzalo Peluffo, José Boggia, Rafael Radi, Homero Rubbo, Andres Trostchansky

**Affiliations:** 1 Departamento de Bioquímica and Center for Free Radical and Biomedical Research, Facultad de Medicina, Universidad de la República, Montevideo, Uruguay; 2 Departamento de Fisiopatología, Hospital de Clínicas, Universidad de la República, Montevideo, Uruguay; University of Rome, ITALY

## Abstract

Nitro-arachidonic acid (NO_2_-AA) is a cell signaling nitroalkene that exerts anti-inflammatory activities during macrophage activation. While angiotensin II (ANG II) produces an increase in reactive oxygen species (ROS) production and mitochondrial dysfunction in renal tubular cells, little is known regarding the potential protective effects of NO_2_-AA in ANG II-mediated kidney injury. As such, this study examines the impact of NO_2_-AA on ANG II-induced mitochondrial dysfunction in an immortalized renal proximal tubule cell line (HK-2 cells). Treatment of HK-2 cells with ANG II increases the production of superoxide (O_2_^●-^), nitric oxide (^●^NO), inducible nitric oxide synthase (NOS2) expression, peroxynitrite (ONOO^-^) and mitochondrial dysfunction. Using high-resolution respirometry, it was observed that the presence of NO_2_-AA prevented ANG II-mediated mitochondrial dysfunction. Attempting to address mechanism, we treated isolated rat kidney mitochondria with ONOO^-^, a key mediator of ANG II-induced mitochondrial damage, in the presence or absence of NO_2_-AA. Whereas the activity of succinate dehydrogenase (SDH) and ATP synthase (ATPase) were diminished upon exposure to ONOO-, they were restored by pre-incubating the mitochondria with NO_2_-AA. Moreover, NO_2_-AA prevents oxidation and nitration of mitochondrial proteins. Combined, these data demonstrate that ANG II-mediated oxidative damage and mitochondrial dysfunction is abrogated by NO_2_-AA, identifying this compound as a promising pharmacological tool to prevent ANG II–induced renal disease.

## Introduction

Nitric oxide (^●^NO)-derived species react with unsaturated fatty acids to yield a variety of bioactive molecules that participate in cell signaling [1,2]. Nitro-fatty acids (NO_2_-FA) have been detected and quantified in cellular and animal models of disease [3,4]; yet, the mechanisms of fatty acid nitration *in vivo* remains unknown [5]. Arachidonic acid (AA) can be nitrated to form a nitroalkene, nitro-arachidonic acid (NO_2_-AA) which exhibits anti-inflammatory actions. For example, NO_2_-AA decreases inducible nitric oxide synthase (NOS2) expression in activated macrophages as well as diminishes secretion of pro-inflammatory cytokines [6]. In addition, we reported that NO_2_-AA is an irreversible inhibitor of prostaglandin endoperoxide H synthase (PGHS) [7] which may contribute to the physiological resolution of inflammatory responses [8]. The formation of superoxide radicals (O_2_^●-^) is also modulated by NO_2_-AA via alteration of NAD(P)H oxidase (NOX) in both activated macrophages and in animal models of inflammation [8].

Angiotensin II (ANG II) is a peptide hormone with a dual role in physiological (blood pressure control and sodium homeostasis) and pathophysiological conditions (pro-inflammatory agent) [9]. The role of ROS in ANG II-induced endothelial dysfunction, cardiovascular and renal remodeling, inflammation and fibrosis has been well documented including increased generation of intracellular ROS and activation of redox-sensitive signaling cascades are seminal events of ANG II action [10]. Specific, molecular mechanisms of ANG II pathophysiological activity involve the stimulation of NOXs thus elevating intracellular O_2_^●-^ production [11,12]. In addition, ANG II promotes endothelial NOS (NOS3) uncoupling [13] which also enhancing increasing O_2_^●-^ generation in cell type-specific manner [14]. Therefore, when ^●^NO production is concomitantly stimulated [15] the formation of the potent oxidant, peroxynitrite may ensue resulting in overt damage [10,16]. Furthermore, ANG II increases the production of mitochondrial ROS while the inhibition of ANG II-derived effects improves mitochondrial function [17]. Interestingly, the over-expression of mitochondrial thioredoxin 2 or mitochondrial superoxide dismutase attenuates ANG II-induced hypertension, which demonstrates the importance of mitochondrial ROS in ANG II-mediated cardiovascular diseases [18].

ANG II induces excessive ROS production and mitochondrial dysfunction, which eventually leads to apoptosis and necrosis of the renal tubular cells [19]. Systemic administration of nitroalkenes such as nitro-oleic acid (NO_2_-OA) in an animal model of hypertension results in inhibition of ANG II type 1 receptor (AT-1)-dependent vasoconstriction and diminution of overall ANG II-induced damage [20]. However, less is known regarding the capacity of NO_2_-AA to protect against ANG II-mediated damage as its biological responses may differ from other nitroalkenes [8]. To analyze the effects of NO_2_-AA and the mechanisms supporting the observed responses, immortalized renal proximal tubule HK-2 cells were treated with ANG II as a model of renal injury and the response to NO_2_-AA treatment was assessed. The overarching aim of this study is to determine if NO_2_-AA treatment results in diminution of O_2_^●-^, ^●^NO and ONOO^-^ production thus preventing mitochondrial oxidative dysfunction in HK-2 cells.

## Materials and Methods

### Materials

Nitro-arachidonic acid was synthesized as previously described [6]. Fluorescein-boronate (Fl-B) was synthesized in our laboratory as in [21] with minor modifications of the borylation reaction, where dioxane was used as the solvent and heated under reflux for 6 h. Peroxynitrite was synthesized from sodium nitrite and H_2_O_2_ using a quenched-flow reactor as previously [22,23]. Rabbit polyclonal antibody against nitro-tyrosine was produced and purified in our laboratory as described [24]. All other reagents were from Sigma Chemical Co (Saint Louis, MO) unless otherwise specified.

### Cell culture

Immortalized human renal proximal tubule cell line (HK-2) was purchased from American Type Culture Collection—ATCC—#2190; Rockville, MD, USA. The cells were grown in DMEM/ Ham's F-12 (Invitrogen-Gibco, Grand Island, NY, USA) 1:1, supplemented with 10% fetal bovine serum (FBS), penicillin (100 units/mL) and streptomycin (100 μg/mL), and maintained at 37°C in humidified air with 5% CO_2_. Cells medium was changed every 72 hours and cells sub cultivated via trypsinization when they were near 80% confluence. Confluent HK-2 cells were incubated with serum-free DMEM/F-12 for 24 h before treatment [25]. Cells were incubated with vehicle (methanol), AA (10 μM) or NO_2_-AA (5–10 μM) for 30 min. In all conditions, methanol did not exceed 0.5%. After washing twice with Dulbecco’s phosphate buffered saline (DPBS), cells were treated with human ANG II (0.1 μM) in serum- free DMEM/F-12 medium for 3 h for mitochondrial function analysis, and 16 h for western blot analysis. Cells were washed twice with DPBS, scraped with 1 mL DPBS and centrifuged to perform the experiments.

### Detection of superoxide by HPLC

HK-2 cells were exposed to 10 μM dihydroethidium (DHE) (Invitrogen) and simultaneously treated with ANG II for 3 h. Cells were centrifuged and the resulting cell pellet stored at -80°C until HPLC analysis. After thawing, the cell pellet was resuspended in DPBS with 0.1% Triton X-100 and cells lysed by aspirating and dispensing back of the suspension using a 1 mL insulin syringe with a needle at least 50 times. Then, an extraction with 600 μL of 1-butanol and vortex for 1 min was done. The mixture was centrifuged 13000 x g for 10 min at 4°C and the upper phase (butanol phase) was removed, dried with vacuum (RapidVap, Labconco), and finally resuspended in mobile phase [26,27]. All steps were done in darkness until analysis. Hydroxyethidium (2-OH-E^+^) and ethidium (Et^+^) products were separated on HPLC system (Agilent Technologies 1200 series) equipped with fluorescence detector. 100 μl of sample was injected into the HPLC system with a stationary phase C18 reverse-phase column (Supelco, 250 mm x 4.6 mm) and the mobile phase corresponded to solvent A: H_2_O/0.1% TFA, solvent B: Acetonitrile (CH_3_CN) 0.1% TFA. The oxidation products were detected at λ_em_ = 595 nm and λ_exc_ = 510 nm, and separated by a linear increase in CH_3_CN concentration as described [26] with minor modification. Either NO_2_-AA or AA was pre-incubated with the cells 30 min prior to activation with ANG II.

### Nitric Oxide production

⋅NO production was determined by detecting the accumulated end product of NO metabolism (nitrite (NO_2_^-^)) with the Griess method [28]. HK-2 cells were treated with ANG II in the presence and absence of NO_2_-AA as before; the culture media separated and the NO_2_^-^ content was determined in the culture supernatant at 570 nm in an UV- Visible spectrophotometer (Varian, Cary 50 Tablet). The participation of NOS was determined by pre-incubating the cells under similar conditions than NO_2_-AA with 1 mM of the⋅NO inhibitor L-N^G^-Nitroarginine methyl ester (L-NAME). Controls with AA were also included.

### Peroxynitrite detection

Peroxynitrite formation was detected by using the Fl-B probe. HK-2 cells were exposed to 30 μM Fl-B, treated with ANG II as previously and kept in the dark until analysis. Then, cells were treated as explained above and the resulting pellet was resuspended in 1 mL DPBS. Fluorescence was analyzed in a FACS Calibur (BD Biosciences) (λexc = 492 nm, λem = 515 nm) [21]. Peroxynitrite formation with cells pre-incubated with NO_2_-AA without activation with ANG II was performed as a control.

### Oxygen consumption

Cell respiration was evaluated using Oxygraph 2 K (Oroboros Instruments Corp). Oxygen consumption was recorded at 37°C in intact cells. The rate of oxygen consumption was calculated by means of the equipment software (DataLab) and was expressed as pmol of O_2_·s^−1^·mL^−1^. For HK-2 respiration, cells were resuspended at 1×10^6^ cells/mL in culture medium.

Baseline measurements of mitochondrial oxygen consumption was performed at the beginning of the assay followed by the sequential addition of an ATP synthase inhibitor (oligomycin), an uncoupler of oxidative phosphorylation (FCCP), and an inhibitor of Complex III (antimycin A). The cells were first titrated with 0.5–4 μM FCCP and 0.5–2.5 μM oligomycin. For experimental purposes, oligomycin final concentration was 2 μM, were it exerted maximum inhibition of oxygen consumption. The maximum oxygen consumption rate was observed at 1 μM FCCP. Thus, 2 μM oligomycin and 1 μM FCCP were used for the following experiments. Non-mitochondrial oxygen consumption rate was determined after the addition of 2.5 μM antimycin A (antimycin resistant oxygen consumption); this value was subtracted from all other values before calculating the reported respiratory parameters. Respiratory parameters were determined as: *i)* basal respiration, baseline; *ii)* respiration driving proton leak, oxygen consumption after the addition of 2 μM oligomycin; *iii)* maximum respiration, after 1 μM FCCP addition; *iv)* cell respiratory control ratio (RCR), maximum respiration of FCCP/respiration driving proton leak; *v)* maximum respiration rate, maximum respiration in the presence the FCCP per 10^6^ cells; *vi)* spare respiratory capacity, maximum respiration/ basal respiration [29,30, 31]

### Mitochondria purification

When trying to obtain mitochondria from HK-2 cells, the yields were very low and the amounts were not enough to perform mechanistically approaches with them. Thus, we performed mitochondrial isolation from rats using kidney as a source to maintain the same tissue. Mitochondria were isolated from rat kidney homogenates and were prepared by differential centrifugation as described previously [32] with all procedures approved by the local authorities (Comisión Honoraria de Experimentación Animal). Briefly, rats were anesthetized and kidney was removed and washed extensively, minced, and homogenized with a tissue grinder. Tissue fragments were disrupted using a Potter-Elvehjem homogenizer in buffer containing 0.3M sucrose, 5 mM morpholinepropanesulfonicacid (MOPS), 5 mM potassium phosphate, 1mM EGTA, and 0.1% BSA. Then, homogenized tissue was centrifuged at 1500 x g, and mitochondria were isolated from the supernatant by centrifugation at 11500 x g. Mitochondrial pellets were resuspended in minimal volume of homogenization buffer. Protein concentration was determined using Bradford [33].

### Kidney mitochondrial exposure to peroxynitrite

Isolated mitochondria were pre-incubated with AA or NO_2_-AA (1, 5 and 10 μM) for 5 min at 25°C. Lipid enrichment of mitochondrial membranes was determined by liquid chromatography- tandem mass spectrometry (LC-MS/MS). Briefly, mitochondria (0.2 mg/mL), after incubation with the lipids and an internal standard, were centrifuged and the pellet extracted and analyzed in the negative ion mode as previously described [6]. The recovery of AA or NO_2_-AA from enriched mitochondria was more than 90%, corresponding to 45.6 ± 4.1 nmol/mg protein in the case of the highest nitroalkene concentration used. Enriched kidney mitochondria was treated with a bolus of peroxynitrite (0.5 mM) for 5 min to analyze enzymatic activities as well as and western blot analysis. According to previous reported work from our lab [34], bolus addition of 0.5 mM peroxynitrite exerts similar extents of inhibition of mitochondrial complexes compared with a continuous flow of the oxidant during 10 minutes. This flow corresponds to a steady state of 1.3 μM peroxynitrite maintained in 10 min, thus allowing us the use of the bolus addition in our experimental conditions.

### Enzymatic assays

ATP synthase (ATPase) activity was determined by monitoring NADPH oxidation at 340 nm (e = 6.22 mM^-1.^ cm^-1^) in a coupled reaction of pyruvate kinase and lactate dehydrogenase [35].The reaction mixture contained treated and non-treated mitochondria, 2.5 mM ATP, 50 mg/mL pyruvate kinase, 50 mg/mL lactate dehydrogenase in 50 Mm Hepes, pH 8 [32]. Succinate dehydrogenase (SDH) was measured spectrophotometrically in the presence of 20 mM 2,6-dichlorophenol indophenol (DCPIP), 15 mM succinate, and 2 mM potassium cyanide (KCN). Reduction of DCPIP was followed at 600 nm (ε = 20.5 mM^-1.^ cm^-1^) [36].

### Derivatization of protein carbonyls for western blot

Two volumes of treated mitochondria were mixed with 1 volume of 24% SDS. Then 1 volume of 20 mM dinitrophenylhidrazyne (DNPH) in 20% TFA was added to the mixture which was then incubated at room temperature for 15 min. Immediately following derivatization, the sample was neutralized and prepared for final gel loading by adding 1/3 volume of Tris/glycerol with 2-mercaptoethanol. Then samples were separated by SDS–PAGE and transferred to PVDF membranes for western blot. As a control, same samples were derivatized in TFA solution without DNPH.

### SDS-PAGE and western blot

Proteins were separated by 10% SDS–PAGE as reported [37]. Gels were transferred to PVDF membranes for protein carbonyl and uNOS immunodetection or nitrocellulose membrane for nitrotyrosine immunodetection. Proteins were electroblotted by a semi-dry Trans-Blot cell. Membranes were incubated with a polyclonal antibody against DNP diluted 1:5000, uNOS diluted 1:1000 and nitrotyrosine diluted 1:1000. For PVDF membranes, immunoreactive bands were detected using a photographic film (Hyperfilm, Amersham Pharmacia Biotech) with an enhanced chemiluminescence kit (Immun-Star ChemiluminescentKit, Bio-Rad, Hercules, CA, USA) and for nitrocellulose membrane, the LiCOR Odyssey system was used for immunodetection.

### Statistical analysis

Experiments were performed at least three times on independent days. Data showed correspond to the mean ± standard error of mean (SEM), unless otherwise noted. Data was analyzed by ANOVA with Student-Newman-Keuls post hoc comparisons. Results were considered significant at p<0.05.

## Results

### NO_2_-AA reduces O_2_⋅^-^production in ANG II-stimulated HK-2 cells

**Fig 1:**
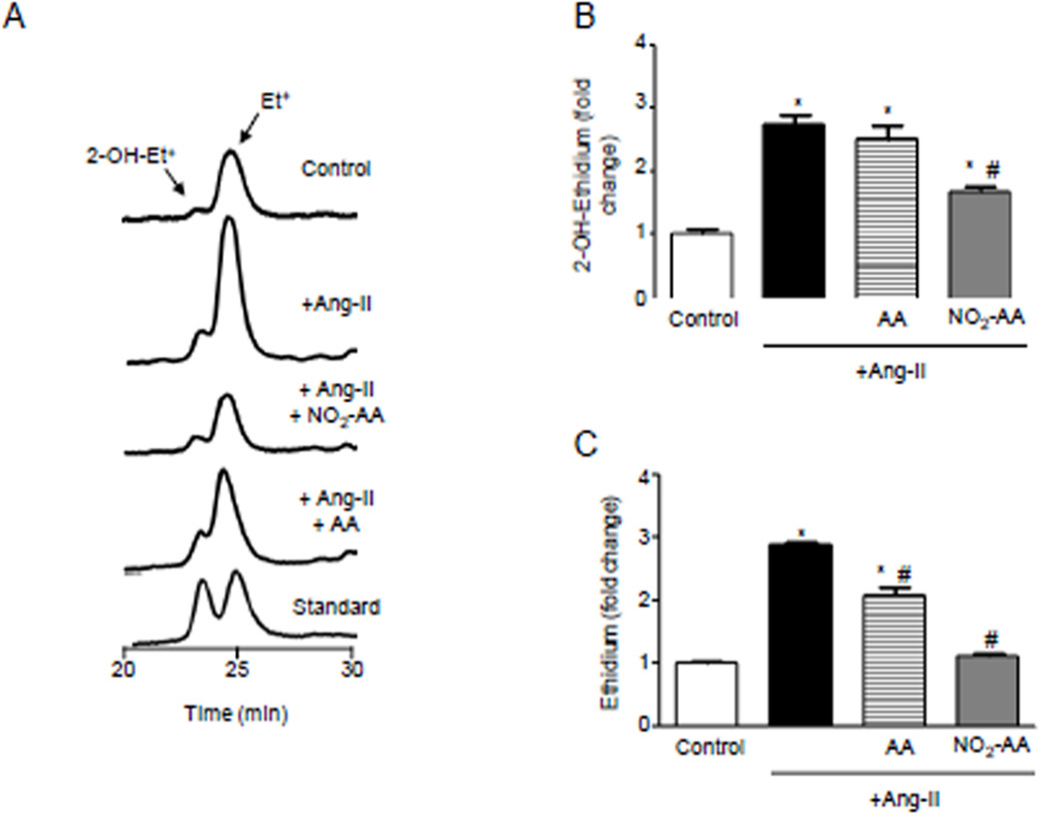
ANG II-mediatedO2.-production is inhibited by NO_2_-AA. (A) Dehydroethydium (DHE) oxidation as an index of O_2_^.-^ production in ANG II-stimulated HK-2 cells (1x10^6^ cells) was determined by HPLC with fluorescence detection (λ_em_ = 595 nm, λ_exc_ = 510 nm). Cells were preincubated 30 min with vehicle, 10 μM NO_2_-AA or 10 μM AA and then exposed to DHE in simultaneous with 0.1 μM ANG II for 3 h. Quantitative analysis, expressed as the mean fold change than control condition without ANG II stimulation ± SEM, *n* = 3 of 2-OH-Et^+^ (B) and Et^+^ (C) are shown. *, # correspond to significant data relative to control and ANG II-treated cells, respectively (p<0.05).

ANG II leads to O_2_^.-^ production as shown by the HPLC profile and the resultant 1.7-fold increase in 2-OH-Et^+^ formation in HK-2 cells compared to untreated controls (Fig 1A and 1B). In parallel, HK-2 cells were pre-incubated with either 10 μM NO_2_-AA or AA and then exposed to DHE simultaneously with 0.1 μM ANG II for 3 h. After treatment with ANG II, no changes were observed in 2-OH-Et^+^ and Et^+^ levels in cells incubated with AA (Fig 1A–1C). Preincubation with NO_2_-AA decreased ~40% and ~50% 2-OH-Et^+^ and Et^+^ formation, respectively (Fig 1B and 1C).

### Decrease in ^●^NO production in ANG II- stimulated HK-2 cells by NO_2_-AA

**Fig 2:**
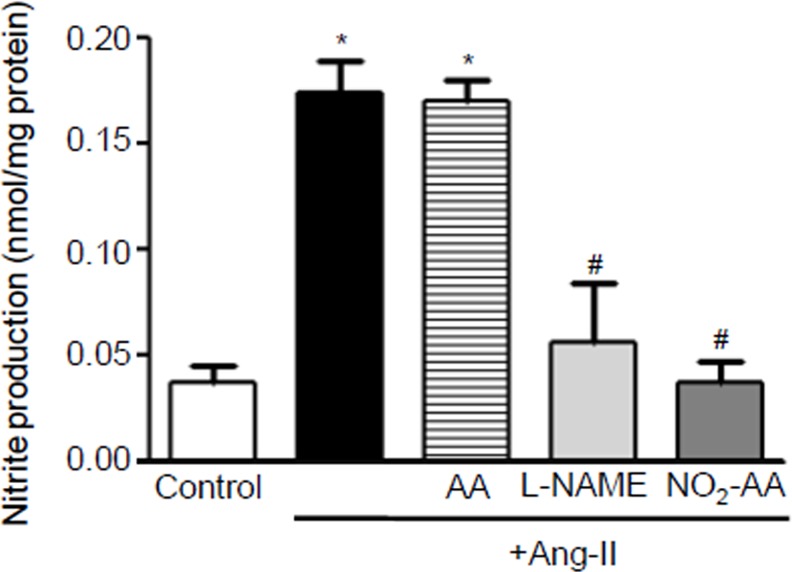
⋅NO production is decreased in ANG II- activated HK-2 cells by NO_2_-AA. Nitrite production was determined in the culture supernatant at 570 nm. Cells were preincubated 30 min with vehicle, 10 μM NO_2_-AA, 10 μM AA or 1 mM L-NAME and then exposed to 0.1 μM ANG II for 3 h, and ⋅NO formation analyzed as explained in Materials and Method section. *, # express significant differences respect to control and ANG II-treated cells, respectively (p<0.05).

Nitric oxide production following 3 h of cell activation with ANG II was determined by detecting NO_2_^-^ levels in the culture media by the Griess reaction [28] (Fig 2). Nitrite production was increase by ANG II stimulation in HK-2 cells when compared to untreated cells (Fig 2). When cells were pre-incubated for 30 min with 10 μM NO_2_-AA prior to ANG II stimulation, NO_2_^-^ production was decreased by ~80% when compared to ANG II alone. As expected, native AA did not alter NO_2_^-^ production (Fig 2). Moreover, ANG II-dependent NOS activation and subsequent NO_2_^-^ accumulation was abrogated by the NOS inhibitor L-NMMA (Fig 2).

### NO_2_-AA inhibited ANG II-induced NOS expression

**Fig 3:**
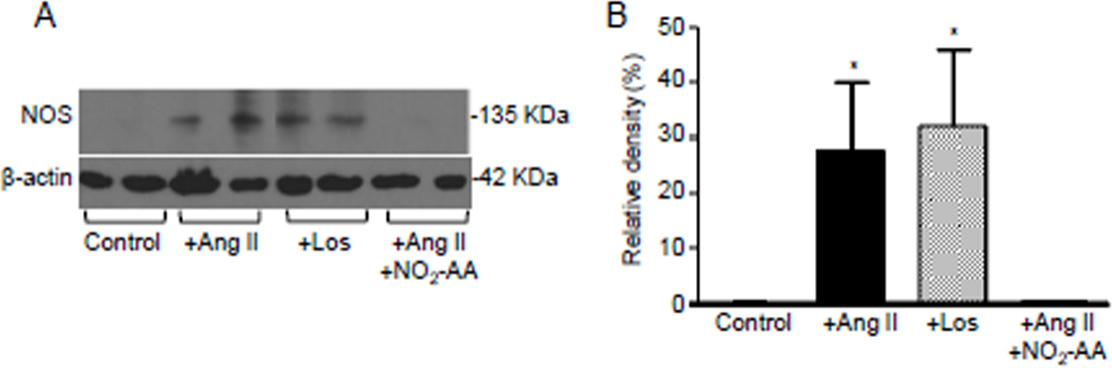
NO_2_-AA decrease NOS expression in ANG II stimulated HK-2 cells. (A)Western blot of NOS expression in ANG II-treated HK-2 cells (1x10^6^ cells) pre-incubated 30 min with vehicle, Losartan or NO_2_-AA was analyzed. (B)Densitometric analysis of the bands was performed and the % of the relative density of NOS to β-actin of the observed bands was plotted as the mean ± SD, n = 3. * express significant differences relative to either control or NO_2_-AA treated cells (p<0.05).

The three NOS isoenzymes are expressed in the kidney, with NOS2 and NOS3 being present in the proximal tubule [14]. Following 16 h of activation with ANG II a significant increase of NOS protein levels was observed (Fig 3). This NOS induction was prevented by NO_2_-AA, reaching control condition levels (Fig 3). Losartan, an AT1 receptor antagonist, had no effect on ANG II-mediated NOS expression (Fig 3) suggesting that induction of NOS by ANG II in HK-2 cells is not due to an AT1 receptor-mediated mechanism.

### Peroxynitrite production in HK-2 cells is inhibited by NO_2_-AA

**Fig 4:**
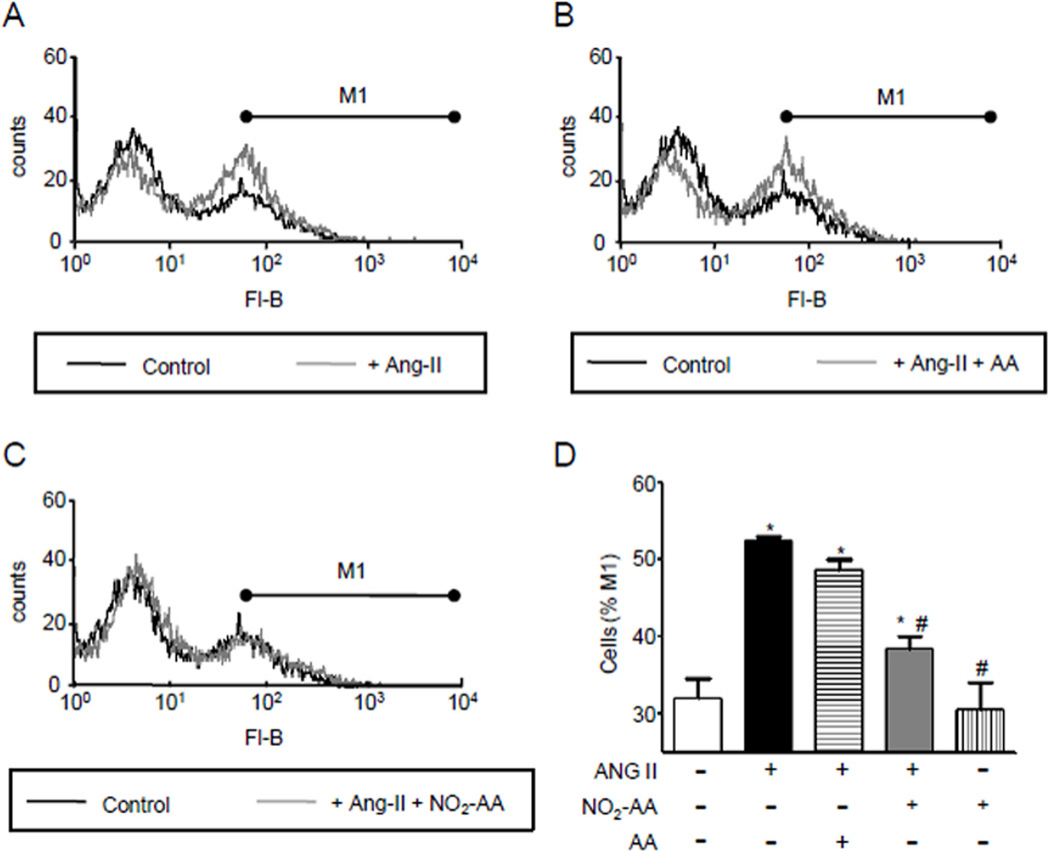
Modulation of ANG II- mediated peroxynitrite production by NO_2_-AA_._ HK-2 cells (1x10^6^ cells) were treated as previously and exposed to 30 μM Fl-B for 3 h. The fluorescence of Fl-B was followed by flow cytometry. Representative histograms are shown for controls compared to the ANG II stimulated cells in the absence (A) or presence of 10 μM AA (B) or 10 μM NO_2_-AA (C). (D) Quantitative analysis of the histograms was performed determining a M1 region that corresponded to the cell population that exhibits high fluorescence due to Fl-B oxidation by peroxynitrite. A control with the nitroalkene in the absence of stimulation with ANG II was included as a control. *, # express significant differences respect to control and ANG II-treated cells, respectively (p<0.05).

As shown previously, NO_2_-AA inhibited O_2_^●-^ and ^●^NO production; the precursors of ONOO^-^. In the present study we evaluated the formation of ONOO^-^ upon ANG II stimulation using novel boronate compounds (Fig 4). Peroxynitrite oxidizes the boronate probe to yield the hydroxylated fluorescent compound that can be assessed by flow-cytometry. Following 3 h of ANG II exposure cells demonstrated an increase in fluorescence due to Fl-B oxidation by ONOO^-^ compared to control conditions (Fig 4A and 4D). When cells were pre-incubated for 30 min with 10 μM NO_2_-AA, ONOO^-^ levels decreased ~40% when compared to ANG II alone (Fig 4D). Exposure to native AA did not alter ANG II-mediated ONOO^-^ formation (Fig 4D). As a control, non-stimulated cells pre-incubated with NO_2_-AA demonstrated similar fluorescence than controls (Fig 4D).

### NO_2_-AA protects mitochondrial function in ANG II stimulated HK-2 cells

**Fig 5:**
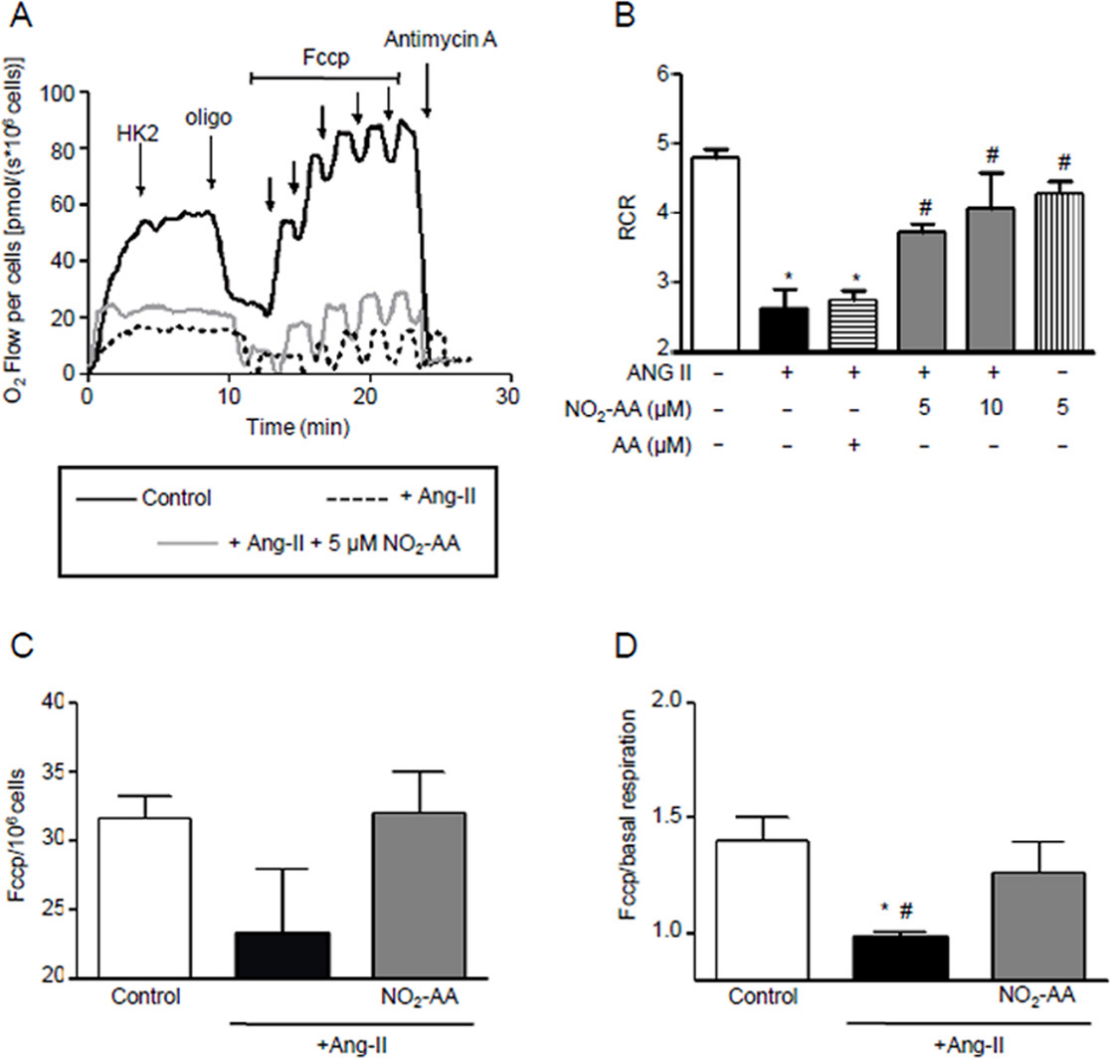
NO_2_-AA improves mitochondrial function in ANG II stimulated HK-2 cells. (A)HK-2 cells (1x10^6^ cells, black line) were pre-incubated with vehicle, 5 or 10 μM NO_2_-AA or 10 μM AA and then treated with 0.1 μM ANG II for 3 h.Oxygen consumption was recorded at 37°C in intact cells using high resolution respirometry (OROBOROS Oxygraph-2K). Arrows indicate steps in the titration regime, inducing the following respiratory states: Oligomycin, inhibition of ATP syntase; FCCP, maximal stimulation by uncoupling of oxidative phosphorylation, and antimycin A, inhibition of complex III. Respiratory control ratio (RCR) values(B), maximal respiratory rate (C) and spare respiratory capacity(D) were determined as explained in Methods section and plotted as the mean ± SEM, *n* = 4. * p<0.05 relative to control cells; # p<0.05 relative to ANG II-treated cells.

High-resolution respirometry showed that ANG II significantly alters mitochondrial function. Cells demonstrated characteristic oxygen consumption rates at basal conditions as well as upon exposure to oligomycin, FCCP and antimycin A (Fig 5A). When the cell respiratory control ratio (RCR) was analyzed, ANG II-treated cells showed a decrease of 50% versus non-treated cells. This parameter was improved by exposure to NO_2_-AA (both 5 μM and 10 μM) while exposure to native AA produced no observable effect (Fig 5B). Moreover, NO_2_-AA had no effect on mitochondrial function in the absence of ANG II stimulation (Fig 5B). Basal oxygen consumption rate was significantly protected in the presence of NO_2_-AA (Fig 5A). The presence of NO_2_-AA showed no effect on the maximum respiratory rate (Fig 5C). Finally, reserve respiratory capacity was protected by NO_2_-AA (Fig 5D).

### NO_2_-AA protects kidney mitochondria from peroxynitrite damage

**Fig 6:**
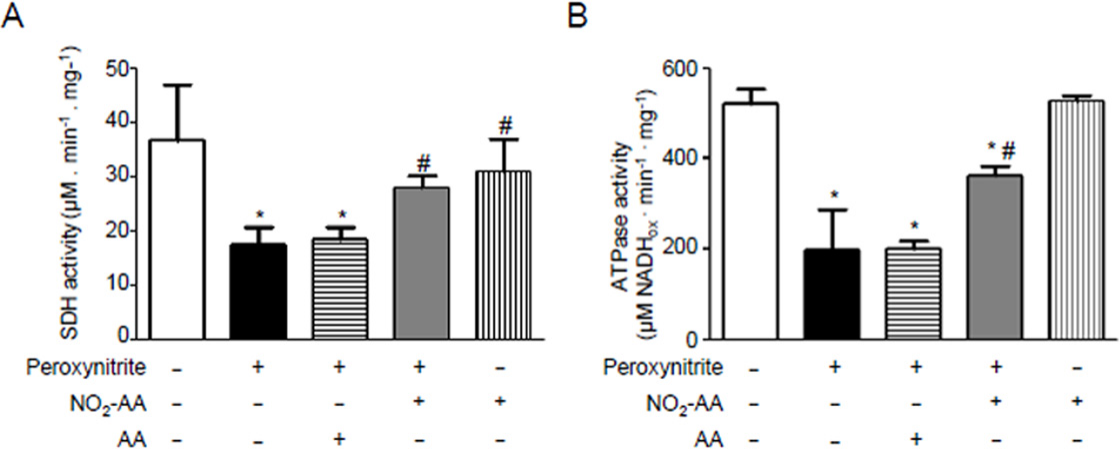
NO_2_-AA spares SDH and ATPase in kidney mitochondria. Isolated mitochondria, enriched with either AA or NO_2_-AA as described in methods, were exposed to peroxynitriteand both SDH(A) and ATPase(B) specific activities determined. Results are representative of three independent experiments and correspond to the mean ± SD, n = 3. Controls of NO_2_-AA addition in the absence of peroxynitrite addition were included for both complexes activities. * p<0.05 data relative to control mitochondria; # p<0.05 relative to peroxynitrite-treated mitochondria.

**Fig 7:**
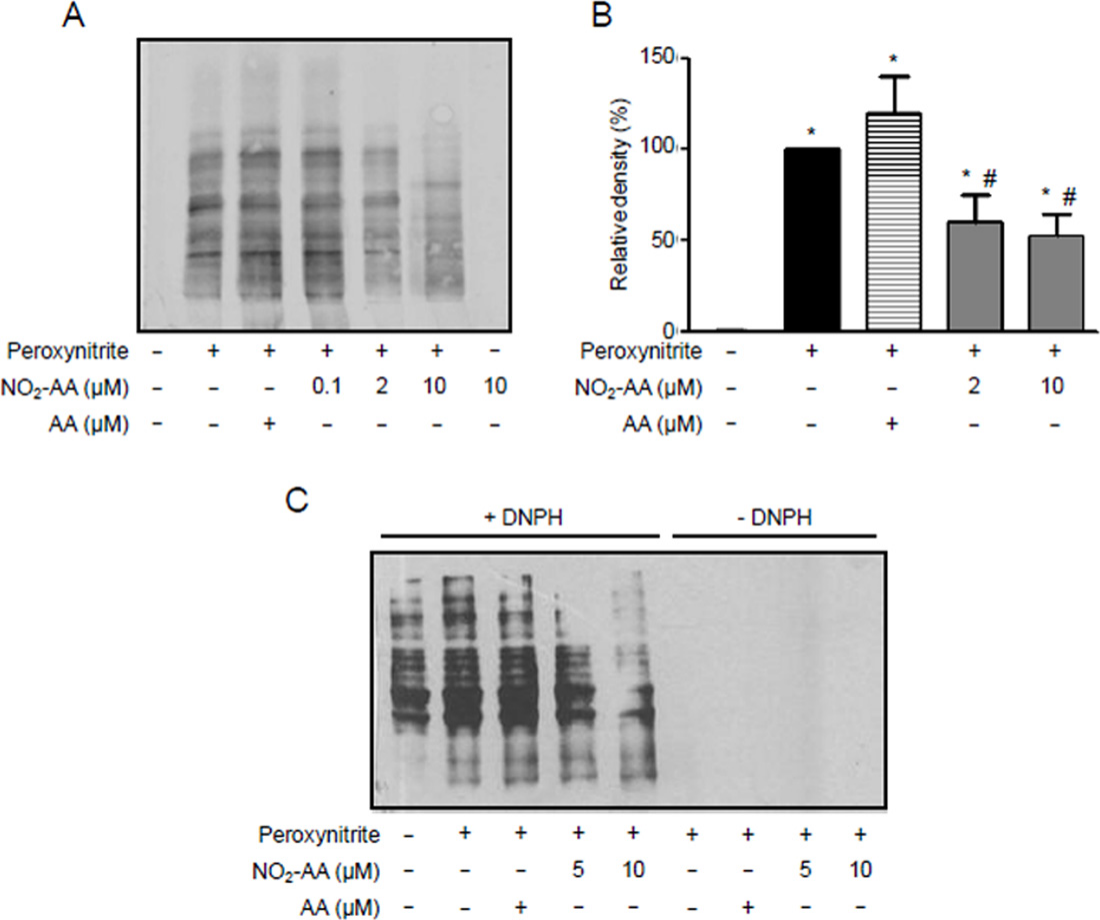
Peroxynitrite- mediated mitochondrial protein oxidation is inhibited by NO_2_-AA. (A) Isolated kidney mitochondria were preincubated with vehicle, AA or NO_2_-AA with peroxynitriteas previously.Mitochondrial proteins were separated in SDS-PAGE and transferred to nitrocellulose membrane for nitrotyrosineimmunodetection with a polyclonal antibody diluted 1:1000. (B) Densitometry of total inmunoreactive bands staining was performed and shown as mean ± SEM, n = 4. * p<0.05 relative to untreated mitochondria; # p<0.05 relative to peroxynitrite-treated mitochondria (C). 10 mg/mL of treated mitochondria were derivatized with DNPH, separated by SDS–PAGE and transferred to PVDF membranes for western blot and incubated with a polyclonal antibody against DNP diluted 1:5000 for protein carbonyls formation. Controls without DNPH derivatization were included.

To identify potential targets of ANG II-derived reactive species, experiments were performed using isolated rat kidney mitochondria. The rationale to use isolated mitochondria belongs from the low yields of mitochondria obtained when using HK-2 cells; in addition, we decided to use isolated mitochondria from rat kidney homogenates to maintain the same tissue origin. Mitochondria enriched with either AA or NO_2_AA were treated with ONOO^-^, in accordance to previous work from our group [38], to analyze modifications on the activity of the oxidative phosphorylation complexes (Fig 6) as well as protein oxidation levels (Fig 7). Complex II (SDH, Fig 6A) as well as ATPase (Fig 6B) was inhibited by ONOO^-^ treatment. In the presence of NO_2_-AA, both SDH (Fig 6A) and ATPase (Fig 6B) activities were improved. As expected, the native AA did not alter the damage induced by ONOO^-^ (Fig 6). Moreover, NO_2_-AA itself did not affect the activity of either complex (Fig 6). Finally, nitration (Fig 7A and 7B) and carbonyl formation (Fig 7C) of mitochondrial proteins were analyzed by western blot as footprints of nitro-oxidative stress. While protein nitration levels were increased in ONOO^—^treated mitochondria from rat kidney (Fig 7A), pre-incubation with NO_2_-AA diminished this process in a dose-dependent manner (Fig 7A and 7B). Similar results were obtained for protein carbonyls (Fig 7C) with the absence of an effect of AA. Again, controls with NO_2_-AA without ONOO^-^ did not exert any effect on mitochondrial protein oxidation (Fig 7A and 7C).

## Discussion

For the last ten years, many studies have addressed the potential use of NO_2_-FAs as pharmacological tools to modulate inflammatory processes in various disease models [39,40,41,42,43]. Previous observations revealed that lipid-derived electrophiles such as NO_2_-OA prevent ANG II-mediated oxidative damage diminishing blood pressure in a mouse model of ANG II-induced hypertension [19]. In addition, the nitroalkene exerts antihypertensive signaling actions by inhibiting soluble epoxide hydrolase (sEH) [19,44]. However, little is known about the biological relevance of NO_2_-AA. Thus, we evaluated the effects of NO_2_-AA on ANG II-treated immortalized HK-2 cells focusing on modulation of cellular damage; in particular the protection of mitochondrial function. Herein, we demonstrate that NO_2_-AA reduced the production of ROS and RNS as a mechanism that supports mitochondrial protection.

We have recently demonstrated that NO_2_-AA decreases O_2_^●-^ formation in activated macrophages by preventing the correct assembly of the active complex of NOX2 [8]. Moreover, in endothelial cells ANG II induces O_2_^●-^ production via activation of membrane-associated NOX [11]. As such, we decided to investigate if ANG II increases oxidant production in HK-2 cells and whether NO_2_-AA can modulate this process. As observed in Fig 1, stimulation with ANG II enhanced O_2_^●-^ production and allied oxidative stress in HK-2 cells. Superoxide reacts with DHE to produce 2-OH-Et^+^, a specific product for detecting O_2_^●-^ in biological systems [27]. In addition, cellular formation of Et^+^ from DHE was analyzed, which can be associated with oxidative stress [26,27] caused by ANG II treatment [11]. HK-2 immortalized cells pretreated with NO_2_-AA showed a decrease in O_2_^●-^ production and oxidative stress when activated with ANG II (Fig 1A and 1B). Pretreatment with native AA had no effect on O_2_^●-^ or allied oxidant production. Overall, ANG II-stimulated elevation in NOX activity was inhibited by NO_2_-AA to provide protection.

In kidney, the three NOS isoforms are located close to the components of the renin-angiotensin system (RAS) as NOS2 and NOS3 are found in the proximal tubule [14]. This may explain the interactions between ANG II and ^●^NO in the kidney. Previous reports in the literature show that ANG II stimulates ^●^NO production in primary human proximal tubular cells in a time-dependent manner [28]. Moreover, NOS inhibitors decreased ^●^NO production in similar conditions [28]. These results are consistent with our observations in HK-2 cells that were stimulated with ANG II (Fig 2). For example, after 3 h of ANG II stimulation, we observed an increase in ^●^NO production. Pre-incubating the cells with either NO_2_-AA or L-NAME produced a decrease in ^●^NO production (Fig 2). On the other hand, the impact of ANG II on the expression of NOS isoforms *in vitro* appears to be cell type-specific [14]. We have demonstrated that NOS expression at longer incubation times (16 h) is induced by ANG II in contrast to unstimulated cells (Fig 3). These results are in accordance with previous data in rats maintained on a low-salt diet that is associated with increased circulating ANG II levels showing more intense immunostaining for NOS2 in kidney tissue [45]. When NO_2_-AA was added in addition to ANG II, NOS levels were similar than those observed in unstimulated cells (Fig 3). Losartan, an AT_1_ receptor antagonist, had no effect on ANG II-mediated NOS expression in HK-2 cells (Fig 3), suggesting that ANG II effects on NOS expression did not involve an AT_1_ receptor mechanism. Moreover, reported data from others groups showed the ability of ANG II to stimulate ^●^NO production in primary cultures of human proximal tubular cells was a process that was not inhibited by either AT_1_ or AT_2_ receptor antagonists [28]. On the other hand, when isolated rat proximal tubules cells were analyzed instead of human cells, ANG II was shown to act via the AT_1_ receptor to activate NO-cGMP pathway [14]. Since NO_2_-AA effects on other cell types are downstream to receptor activation, more work needs to be done in order to determine the exact mechanism of NOS expression due to ANG II in HK-2 cells.

**Fig 8:**
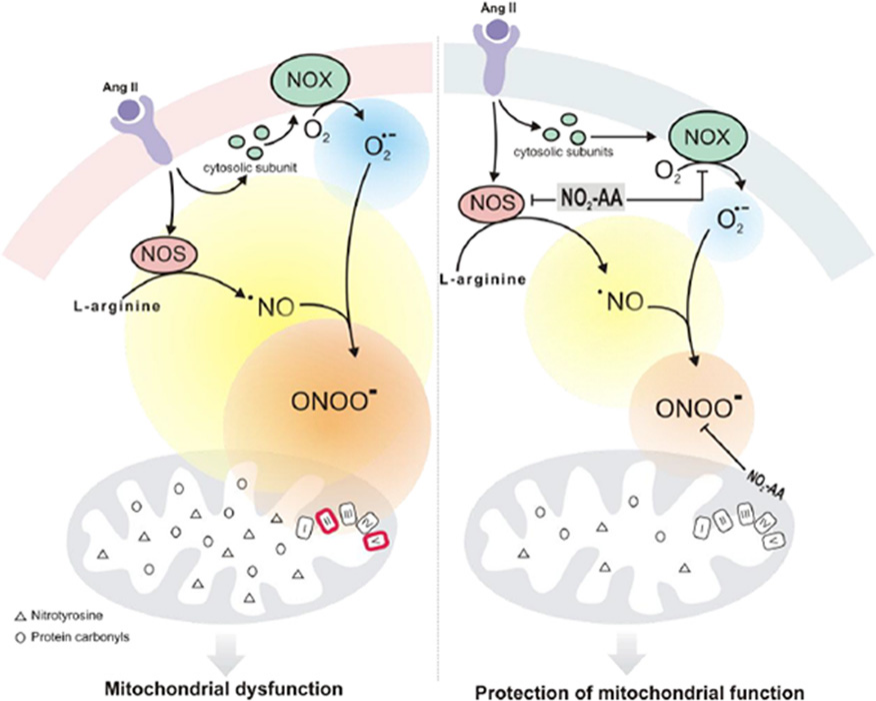
Schematic representation of mitochondrial dysfunction modulation by NO_2_-AA in a cellular model of kidney cells activated by ANG II. On the left of the diagram, the stimulation with ANG II in HK-2 increases the O_2_^.-^ production as well as inducible NOS expression and formation of peroxynitrite. This highly oxidizing molecule produces a decrease in the activities (red line) of the respiratory chain complex SDH (complex II) and ATPase (complex V) as well as increases oxidation and nitration of mitochondrial proteins. Therefore, HK-2 exposed to ANG II exhibited mitochondrial dysfunction. The right side of the diagram represent the modulation of cell damage by the nitroalkene. In the presence of NO_2_-AA a reduction of ANG II-induced HK-2 damage is produced, with lower extents of O_2_^.-^ production as well as lower levels of inducible NOS expression leading to a decrease in peroxynitrite formation. The activities of the respiratory chain complex are restored and NO_2_-AA also prevents oxidation and nitration of mitochondrial proteins. In summary, NO_2_-AA modulates ANG II mediated oxidative damage improving mitochondrial function.

Superoxide and ^●^NO rapidly react to form the potent oxidant and nitrating agent ONOO^-^ [11,15]. When O_2_^●-^ formation increases, ^●^NO induces a prooxidant action by decreasing its bioavailability leading to the formation of peroxynitrite. Since the formation of both precursors were increased by ANG II activation of immortalized HK-2 cells (Figs 1and 2) we focused our studies to determine if the formation of ONOO^-^ is the main intermediate of the observed nitro-oxidative stress. For ONOO^-^ detection, we used boronate-based compounds with a fluorescein moiety covalently attached to the boronic ester (Fl-B). Following reaction with ONOO^-^, the corresponding hydroxylated fluorescent compound is formed and intracellular production can be followed by flow cytometry. Fl-B reacts preferentially with ONOO^-^ due to its most favorable kinetic profile over other relevant nucleophiles. Herein, after stimulation with ANG II we observed an segment of the cell population exhibiting high fluorescence due to Fl-B oxidation confirming ONOO^-^ production (Fig 4A and 4D). Although many reports suggest that ONOO^-^ is formed by ANG II stimulation due to an increase of ROS and ^●^NO as well as nitrotyrosine formation [10,16], our results are the first direct determination of ONOO^-^ formation in ANG II activated cells (Fig 4). ROS and ONOO^-^ production are enhanced by ANG II as well as mitochondrial oxidants depressing mitochondrial energy metabolism. This scenario is associated with mitochondrial dysfunction in the kidney [10,15,46]. When mitochondrial function was measured in intact cells using high-resolution respirometry, ANG II-treated HK-2 cells showed lower RCR compared to unstimulated cells confirming mitochondrial dysfunction (Fig 5A and 5B). Basal oxygen consumption rate was significantly protected in the presence of NO_2_-AA while no effect exerted on maximum respiration rate (Fig 5). Previous reports suggest that mitochondrial dysfunction precedes the emergence of hypertension due to the existence of a causal relationship between a mitochondrial mutation and hypertension [47]. Protection of mitochondria, in our case by NO_2_-AA, can exert modulatory effects on the ANG II-derived inflammatory processes (Fig 8). To validate our hypothesis, we first determined the effect of NO_2_-AA on ONOO^-^ production (Fig 4C and 4D). In the presence of NO_2_-AA, ANG II- activated cells exhibited a reduction on ONOO^-^ formation (Fig 4D). Moreover, the addition of AA did not affect the production of ONOO^-^ by activated cells (Fig 4C and 4D). The presence of NO_2_-AA improved the mitochondrial coupling (RCR) in HK-2 cells, almost restoring the RCR values obtained under control conditions (Fig 5B). Overall, NO_2_-AA displays protective antioxidant effects and preserves HK-2 cells mitochondria favoring the maintenance of its function.

Finally, we decided to elucidate a mechanism (Fig 8) by which ONOO^-^ is responsible for the observed oxidative damage and consequent mitochondrial dysfunction and how therefore, NO_2_-AA exerts its beneficial actions by protecting mitochondrial damage (Figs 6 and 7). Since the yields of mitochondria isolation from HK-2 cells were very low, we decided to use isolated mitochondria from rat kidney homogenates to maintain the same origin. Mitochondria were exposed to bolus addition of ONOO^-^ under experimental conditions that mimic a steady-state concentration of 1.3 μM maintained for 10 minutes [32]. In accordance with previously reports by our group [34,38], ONOO^-^ significantly inactivated SDH and ATPase (Fig 6). In both cases, NO_2_-AA-, but not AA-, diminished the damaged exerted by ONOO^-^ suggesting that the mitochondrial protection observed for NO_2_-AA in intact cells can be due to mitochondrial complex protection (Figs 6 and 8). Importantly, more than 90% of either the nitrated or non-nitrated AA were incorporated to mitochondria as analyzed by LC-MS/MS (data not shown), thus suggesting that the presence of the nitroalkene at the mitochondrial membranes was necessary to exert the observed protection. It has been reported that protein oxidation (*e*.*g*. 3-nitrotyrosine and protein carbonyls) represents footprints of nitro-oxidative damage caused by ONOO^-^ [48]. When mitochondrial proteins were analyzed after ONOO^-^ addition, NO_2_-AA decreased both nitrotyrosine (Fig 7A and 7B) and protein carbonyls (Fig 7C) in a dose-dependent manner.

Since nitroalkenes are endogenously formed at levels ranging from nanomolar to low micromolar and they exhibit important cell signaling actions [3,49], then their use as novel pharmacological tools should be relevant. Of importance to our work, it has been reported that nitroalkenes can be formed endogenously in mitochondria at μM levels under ischemic preconditioning conditions [50]. Thus, the NO_2_-AA concentrations used in our studies, besides being in the pharmacologic, are also in the pathophysiological range affirming the relevance to the reported data in this manuscript. Overall, our results show that NO_2_-AA modulates ANG II-mediated inflammatory damage in HK-2 cells through prevention of ONOO^-^ formation and subsequent mitochondrial dysfunction (Fig 8). The protection of mitochondrial function by NO_2_-AA in a cellular model of renal injury emphasizes the use of nitroalkenes as novel mitochondrial-targeted antioxidants exhibiting potent anti-inflammatory actions.

## Abbreviations

AA: Arachidonic acid
ANG II: Angiotensin II
DCPIP: 2,6-dichlorophenol indophenol
DHE: Dihydroethidium
DNPH: Dinitrophenylhydrozyne
DPBS: Phosphate buffered saline
Et^+^: Ethidium
Fl-B: Fluorescein-boronate
HK-2: Human renal proximal tubular epithelial cell line
KCN: Potassium cyadine
LC-MS/MS: liquid chromatography- tandem mass spectrometry
L-NAME: L-NG-Nitroarginine methyl ester
⋅NO: Nitric oxide
⋅NO_2_: Nitrogen dioxide
NO_2_-: Nitrite
NO_2_-AA: Nitro-arachidonic acid
NO_2_-FA: Nitro-fatty acids
NO_2_-OA: Nitro-oleic acid
NOS2: Inducible nitric oxide synthase
NOS3: Endothelial nitric oxide synthase
NOX: NAD(P)H oxidase
2-OH-Et^+^: 2-hydroxyethidium
O_2_^.-^: Superoxide radical
PGHS-2: Prostaglandin endoperoxide H synthase
RAS: Renin-angiotensin system
RCR: Respiratory control ratio
RNS: Reactive nitrogen species
ROS: Reactive oxygen species
SDH: Succinate dehydrogenase
she: Soluble epoxide hydrolase

## References

[1] Rubbo H, Radi R, Trujillo M, Telleri R, Kalyanaraman B, Barnes S, et al Nitric oxide regulation of superoxide and peroxynitrite-dependent lipid peroxidation. Formation of novel nitrogen-containing oxidized lipid derivatives. J Biol Chem. 1994;269: 26066–26075. 7929318

[2] Schopfer FJ, Cipollina C, Freeman BA. Formation and signaling actions of electrophilic lipids. Chem Rev. 2011;111: 5997–6021. 10.1021/cr200131e 21928855 PMC3294277

[3] Baker PR, Lin Y, Schopfer FJ, Woodcock SR, Groeger AL, Batthyany C, et al Fatty acid transduction of nitric oxide signaling: multiple nitrated unsaturated fatty acid derivatives exist in human blood and urine and serve as endogenous peroxisome proliferator-activated receptor ligands. J Biol Chem. 2005;280: 42464–42475. 16227625 10.1074/jbc.M504212200 PMC2266087

[4] Lima ES, Di Mascio P, Rubbo H, Abdalla DS. Characterization of linoleic acid nitration in human blood plasma by mass spectrometry. Biochemistry. 2002;41: 10717–10722. 12186558 10.1021/bi025504j

[5] Rubbo H. Nitro-fatty acids: novel anti-inflammatory lipid mediators. Braz J Med Biol Res. 2013;46: 728–734. 10.1590/1414-431X20133202 24068188 PMC3854434

[6] Trostchansky A, Souza JM, Ferreira A, Ferrari M, Blanco F, Trujillo M, et al Synthesis, isomer characterization, and anti-inflammatory properties of nitroarachidonate. Biochemistry. 2007;46: 4645–4653. 17373826 10.1021/bi602652j

[7] Trostchansky A, Bonilla L, Thomas CP, O'Donnell VB, Marnett LJ, Radi R, et al Nitroarachidonic acid, a novel peroxidase inhibitor of prostaglandin endoperoxide H synthases 1 and 2. J Biol Chem. 2011;286: 12891–12900. 10.1074/jbc.M110.154518 21266582 PMC3075636

[8] Gonzalez-Perilli L, Alvarez MN, Prolo C, Radi R, Rubbo H, Trostchansky A. Nitroarachidonic acid prevents NADPH oxidase assembly and superoxide radical production in activated macrophages. Free Radic Biol Med. 2013;58: 126–133. 10.1016/j.freeradbiomed.2012.12.020 23318789 PMC3622795

[9] Touyz RM. Molecular and cellular mechanisms in vascular injury in hypertension: role of angiotensin II. Curr Opin Nephrol Hypertens. 2005;14: 125–131. 15687838 10.1097/00041552-200503000-00007

[10] de Cavanagh EM, Inserra F, Ferder L. Angiotensin II blockade: a strategy to slow ageing by protecting mitochondria? Cardiovasc Res. 2011;89: 31–40. 10.1093/cvr/cvq285 20819950

[11] Griendling KK, Ushio-Fukai M. Reactive oxygen species as mediators of angiotensin II signaling. Regul Pept. 2000;91: 21–27. 10967199 10.1016/s0167-0115(00)00136-1

[12] Kimura S, Zhang GX, Nishiyama A, Shokoji T, Yao L, Fan YY, et al Role of NAD(P)H oxidase- and mitochondria-derived reactive oxygen species in cardioprotection of ischemic reperfusion injury by angiotensin II. Hypertension. 2005;45: 860–866. 15824196 10.1161/01.HYP.0000163462.98381.7f

[13] Mollnau H, Wendt M, Szocs K, Lassegue B, Schulz E, Oelze M, et al Effects of angiotensin II infusion on the expression and function of NAD(P)H oxidase and components of nitric oxide/cGMP signaling. Circ Res. 2002;90: E58–65. 11884382 10.1161/01.res.0000012569.55432.02

[14] Millatt LJ, Abdel-Rahman EM, Siragy HM. Angiotensin II and nitric oxide: a question of balance. Regul Pept. 1999;81: 1–10. 10395403 10.1016/s0167-0115(99)00027-0

[15] Pueyo ME, Arnal JF, Rami J, Michel JB. Angiotensin II stimulates the production of NO and peroxynitrite in endothelial cells. Am J Physiol. 1998;274: C214–220. 9458730 10.1152/ajpcell.1998.274.1.C214

[16] Lee DY, Wauquier F, Eid AA, Roman LJ, Ghosh-Choudhury G, Khazim K, et al Nox4 NADPH oxidase mediates peroxynitrite-dependent uncoupling of endothelial nitric-oxide synthase and fibronectin expression in response to angiotensin II: role of mitochondrial reactive oxygen species. J Biol Chem. 2013;288: 28668–28686. 10.1074/jbc.M113.470971 23940049 PMC3789965

[17] Dikalov SI, Nazarewicz RR. Angiotensin II-induced production of mitochondrial reactive oxygen species: potential mechanisms and relevance for cardiovascular disease. Antioxid Redox Signal. 2013;19: 1085–1094. 10.1089/ars.2012.4604 22443458 PMC3771548

[18] Doughan AK, Harrison DG, Dikalov SI. Molecular mechanisms of angiotensin II-mediated mitochondrial dysfunction: linking mitochondrial oxidative damage and vascular endothelial dysfunction. Circ Res. 2008;102: 488–496. 18096818 10.1161/CIRCRESAHA.107.162800

[19] Sun L, Xiao L, Nie J, Liu FY, Ling GH, Zhu XJ, et al p66Shc mediates high-glucose and angiotensin II-induced oxidative stress renal tubular injury via mitochondrial-dependent apoptotic pathway. Am J Physiol Renal Physiol. 2010;299: F1014–1025. 10.1152/ajprenal.00414.2010 20739391 PMC2980400

[20] Zhang J, Villacorta L, Chang L, Fan Z, Hamblin M, Zhu T, et al Nitro-oleic acid inhibits angiotensin II-induced hypertension. Circ Res. 2010;107: 540–548. 10.1161/CIRCRESAHA.110.218404 20558825 PMC2937264

[21] Dickinson BC, Huynh C, Chang CJ. A palette of fluorescent probes with varying emission colors for imaging hydrogen peroxide signaling in living cells. J Am Chem Soc. 2010;132: 5906–5915. 10.1021/ja1014103 20361787 PMC2862989

[22] Trostchansky A, Batthyany C, Botti H, Radi R, Denicola A, Rubbo H. Formation of lipid-protein adducts in low-density lipoprotein by fluxes of peroxynitrite and its inhibition by nitric oxide. Arch Biochem Biophys. 2001;395: 225–232. 11697860 10.1006/abbi.2001.2583

[23] Trostchansky A, Ferrer-Sueta G, Batthyany C, Botti H, Batinic-Haberle I, Radi R, et al Peroxynitrite flux-mediated LDL oxidation is inhibited by manganese porphyrins in the presence of uric acid. Free Radic Biol Med. 2003;35: 1293–1300. 14607528 10.1016/j.freeradbiomed.2003.07.004

[24] Brito C, Naviliat M, Tiscornia AC, Vuillier F, Gualco G, Dighiero G, et al Peroxynitrite inhibits T lymphocyte activation and proliferation by promoting impairment of tyrosine phosphorylation and peroxynitrite-driven apoptotic death. J Immunol. 1999;162: 3356–3366. 10092790

[25] Bryson JM, Coy PE, Gottlob K, Hay N, Robey RB. Increased hexokinase activity, of either ectopic or endogenous origin, protects renal epithelial cells against acute oxidant-induced cell death. J Biol Chem. 2002;277: 11392–11400. 11751868 10.1074/jbc.M110927200

[26] Zhao H, Joseph J, Fales HM, Sokoloski EA, Levine RL, Vasquez-Vivar J, et al Detection and characterization of the product of hydroethidine and intracellular superoxide by HPLC and limitations of fluorescence. Proc Natl Acad Sci U S A. 2005;102: 5727–5732. 15824309 10.1073/pnas.0501719102 PMC556312

[27] Zielonka J, Hardy M, Kalyanaraman B. HPLC study of oxidation products of hydroethidine in chemical and biological systems: ramifications in superoxide measurements. Free Radic Biol Med. 2009;46: 329–338. 10.1016/j.freeradbiomed.2008.10.031 19026738 PMC3375818

[28] McLay JS, Chatterjee PK, Mistry SK, Weerakody RP, Jardine AG, McKay NG, et al Atrial natriuretic factor and angiotensin II stimulate nitric oxide release from human proximal tubular cells. Clin Sci (Lond). 1995;89: 527–531. 8549068 10.1042/cs0890527

[29] Gnaiger E. Mitochondrial Pathways and Respiratory Control. OROBOROS MiPNet Publications 2007; Innsbruck: 96 pp.

[30] Brand MD, Nicholls DG. Assessing mitochondrial dysfunction in cells. Biochem J. 2011;435: 297–312. 10.1042/BJ20110162 21726199 PMC3076726

[31] Choi SW, Gerencser AA, Ng R, Flynn JM, Melov S, Danielson SR, et al No consistent bioenergetic defects in presynaptic nerve terminals isolated from mouse models of Alzheimer's disease. J Neurosci. 2012;32: 16775–16784. 10.1523/JNEUROSCI.2414-12.2012 23175831 PMC3736741

[32] Cassina A, Radi R. Differential inhibitory action of nitric oxide and peroxynitrite on mitochondrial electron transport. Arch Biochem Biophys. 1996;328: 309–316. 8645009 10.1006/abbi.1996.0178

[33] Bradford MM. A rapid and sensitive method for the quantitation of microgram quantities of protein utilizing the principle of protein-dye binding. Anal Biochem. 1976;72: 248–254. 942051 10.1016/0003-2697(76)90527-3

[34] Radi R, Rodriguez M, Castro L, Telleri R. Inhibition of mitochondrial electron transport by peroxynitrite. Arch Biochem Biophys. 1994;308: 89–95. 8311480 10.1006/abbi.1994.1013

[35] Pullman ME, Penefsky HS, Datta A, Racker E. Partial resolution of the enzymes catalyzing oxidative phosphorylation. I. Purification and properties of soluble dinitrophenol-stimulated adenosine triphosphatase. J Biol Chem. 1960;235: 3322–3329. 13738472

[36] Singer TP. Determination of the activity of succinate, NADH, choline, and alpha-glycerophosphate dehydrogenases. Methods Biochem Anal. 1974;22: 123–175. 4155042 10.1002/9780470110423.ch3

[37] Laemmli UK. Cleavage of structural proteins during the assembly of the head of bacteriophage T4. Nature. 1970;227: 680–685. 5432063 10.1038/227680a0

[38] Radi R. Peroxynitrite, a stealthy biological oxidant. J Biol Chem. 2013;288: 26464–26472. 10.1074/jbc.R113.472936 23861390 PMC3772193

[39] Cole MP, Rudolph TK, Khoo NK, Motanya UN, Golin-Bisello F, Wertz JW, et al Nitro-fatty acid inhibition of neointima formation after endoluminal vessel injury. Circ Res. 2009;105: 965–972. 10.1161/CIRCRESAHA.109.199075 19797175 PMC2784279

[40] Cui T, Schopfer FJ, Zhang J, Chen K, Ichikawa T, Baker PR, et al Nitrated fatty acids: Endogenous anti-inflammatory signaling mediators. J Biol Chem. 2006;281: 35686–35698. 16887803 10.1074/jbc.M603357200 PMC2169500

[41] Kelley EE, Baust J, Bonacci G, Golin-Bisello F, Devlin JE, St Croix CM, et al Fatty acid nitroalkenes ameliorate glucose intolerance and pulmonary hypertension in high-fat diet-induced obesity. Cardiovasc Res. 2014;101: 352–363. 10.1093/cvr/cvt341 24385344 PMC3928004

[42] Klinke A, Moller A, Pekarova M, Ravekes T, Friedrichs K, Berlin M, et al Protective effects of 10-nitro-oleic acid in a hypoxia-induced murine model of pulmonary hypertension. Am J Respir Cell Mol Biol. 2014;51: 155–162. 10.1165/rcmb.2013-0063OC 24521348 PMC4091852

[43] Rudolph V, Rudolph TK, Schopfer FJ, Bonacci G, Woodcock SR, Cole MP, et al Endogenous generation and protective effects of nitro-fatty acids in a murine model of focal cardiac ischaemia and reperfusion. Cardiovasc Res. 2010;85: 155–166. 10.1093/cvr/cvp275 19666678 PMC2791055

[44] Charles RL, Rudyk O, Prysyazhna O, Kamynina A, Yang J, Morisseau C, et al Protection from hypertension in mice by the Mediterranean diet is mediated by nitro fatty acid inhibition of soluble epoxide hydrolase. Proc Natl Acad Sci U S A. 2014;111: 8167–8172. 10.1073/pnas.1402965111 24843165 PMC4050620

[45] Tojo A, Madsen KM, Wilcox CS. Expression of immunoreactive nitric oxide synthase isoforms in rat kidney. Effects of dietary salt and losartan. Jpn Heart J. 1995;36: 389–398. 7544416 10.1536/ihj.36.389

[46] de Cavanagh EM, Toblli JE, Ferder L, Piotrkowski B, Stella I, Inserra F. Renal mitochondrial dysfunction in spontaneously hypertensive rats is attenuated by losartan but not by amlodipine. Am J Physiol Regul Integr Comp Physiol. 2006;290: R1616–1625. 16410402 10.1152/ajpregu.00615.2005

[47] Wilson FH, Hariri A, Farhi A, Zhao H, Petersen KF, Toka HR, et al A cluster of metabolic defects caused by mutation in a mitochondrial tRNA. Science. 2004;306: 1190–1194. 15498972 10.1126/science.1102521 PMC3033655

[48] Rubbo H, Radi R. Protein and lipid nitration: role in redox signaling and injury. Biochim Biophys Acta. 2008;1780: 1318–1324. 10.1016/j.bbagen.2008.03.007 18395525

[49] Tsikas D, Zoerner AA, Jordan J. Oxidized and nitrated oleic acid in biological systems: analysis by GC-MS/MS and LC-MS/MS, and biological significance. Biochim Biophys Acta. 2011;1811: 694–705. 10.1016/j.bbalip.2011.06.015 21771665

[50] Nadtochiy SM, Baker PR, Freeman BA, Brookes PS. Mitochondrial nitroalkene formation and mild uncoupling in ischaemic preconditioning: implications for cardioprotection. Cardiovasc Res. 2009;82: 333–340. 10.1093/cvr/cvn323 19050010 PMC2675927

