# Correction: Nitro-Arachidonic Acid Prevents Angiotensin II Induced Mitochondrial Dysfunction in Kidney Proximal Tubular Cells

**DOI:** 10.1371/journal.pone.0154651

**Published:** 2016-04-26

**Authors:** Beatriz Sánchez-Calvo, Adriana Cassina, Natalia Rios, Gonzalo Peluffo, José Boggia, Rafael Radi, Homero Rubbo, Andrés Trostchansky

The following information is missing from the funding section: This work was supported by grants from Fondo Clemente Estable-ANII (FCE_6353) to AT and (FCE_6363) to GP, CSIC-Grupos (Uruguay) to HR and RR. BSC was supported by a postdoctoral fellowship by ANII-Uruguay. NR was partially supported by a fellowship from Sistema Nacional de Becas-ANII.

In Panel B of Fig 1, the x-axis is missing. Please see the corrected Fig 1 here.

**Fig 1:**
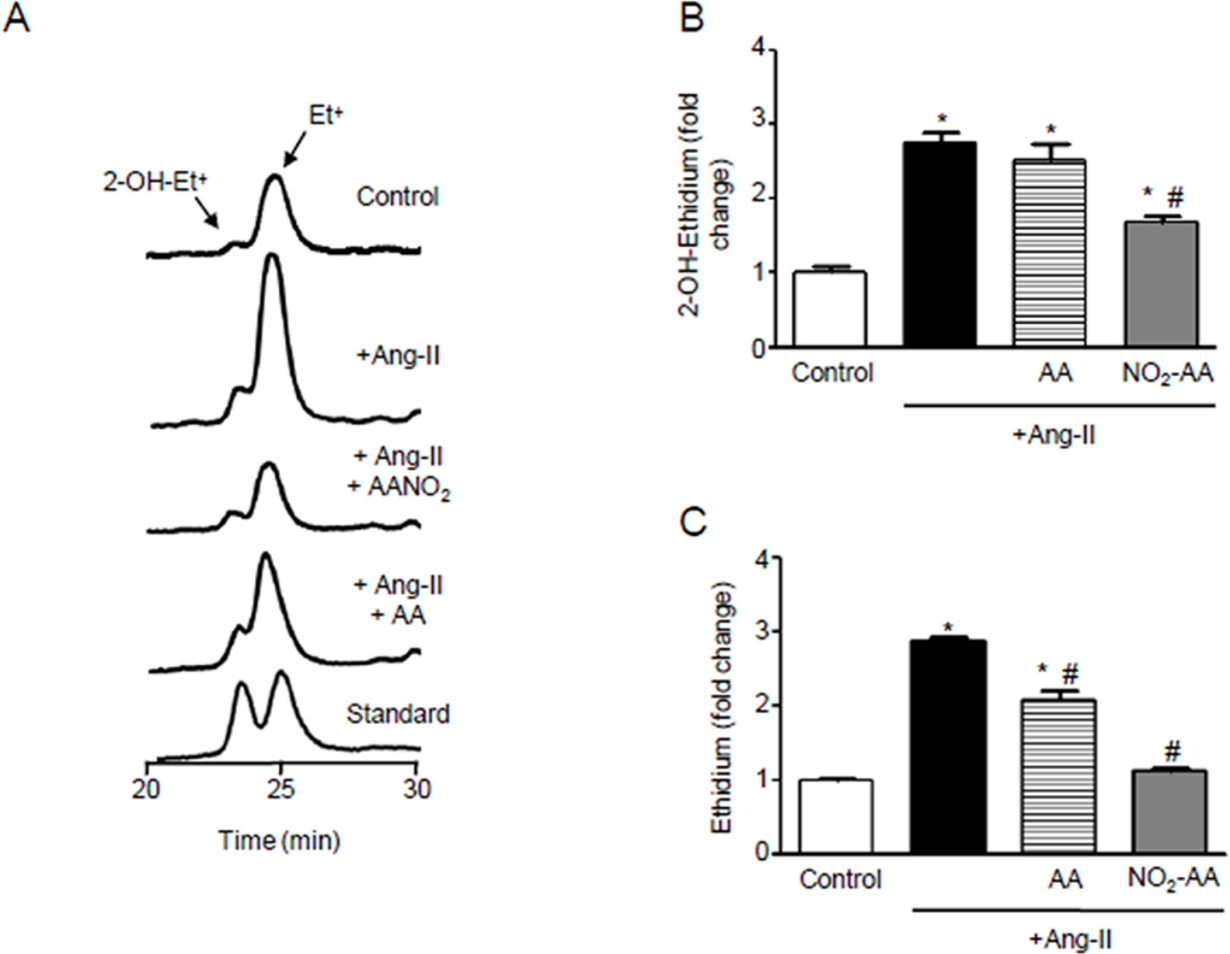
ANG II-mediatedO_2_.-production is inhibited by NO_2_-AA. (A) Dehydroethydium (DHE) oxidation as an index of O_2_.- production in ANG II-stimulated HK-2 cells (1x106 cells) was determined by HPLC with fluorescence detection (λem = 595 nm, λexc = 510 nm). Cells were preincubated 30 min with vehicle, 10 μM NO_2_-AA or 10 μM AA and then exposed to DHE in simultaneous with 0.1 μM ANG II for 3 h. Quantitative analysis, expressed as the mean fold change than control condition without ANG II stimulation ± SEM, n = 3 of 2-OH-Et+ (B) and Et+ (C) are shown. *, # correspond to significant data relative to control and ANG II-treated cells, respectively (p<0.05).
